# Contribution of neurological soft signs’ studies to the understanding of the pathophysiology of schizophrenia

**DOI:** 10.1192/j.eurpsy.2023.2315

**Published:** 2023-07-19

**Authors:** T. Reynolds De Sousa, R. André, F. Novais

**Affiliations:** Psychiatry, Centro Hospitalar Universitário Lisboa Norte, Lisboa, Portugal

## Abstract

**Introduction:**

Neurological soft signs (NSS) have long been described in schizophrenic patients. However, recent studies focusing on first-episode psychosis and at-risk mental states have brought up some aspects that may point to a neurodevelopmental underpinning of the disease.

**Objectives:**

We aimed to review the published literature concerning NSS and psychosis and critically analyze it in regard to how it may constitute a body of evidence favouring the neurodevelopmental hypothesis of schizophrenia.

**Methods:**

We conducted a Pubmed ® research using the following terms “neurological soft signs”, “psychosis”, “psychotic” and “first-episode”.

**Results:**

The studies that have been carried out found a gradation of NSS scores that had its minimum values in healthy controls, intermediate scores in at-risk mental state individuals, and highest scores in first-episode psychosis. NSS correlate with various brain imaging anomalies, which indicates abnormal neurological function. Its scores also correlate with poorer cognitive performance and more prominent negative symptoms in the short- and long-term. Interestingly, patients who have psychotic episodes associated with cannabis use have lower NSS scores than all the other psychotic-illness diagnostic groups.

**Conclusions:**

NSS might thus translate a neurological dysfunction that exists previous to the psychotic break and is a measure of one’s vulnerability to psychosis. These results point to the existence of two distinct groups: one that has high NSS scores and therefore a high genetic vulnerability, needing little contribution of environmental factors to manifest a psychotic episode; and another one with low NSS scores, a smaller genetic vulnerability and a greater role played by environmental influences.

**Disclosure of Interest:**

None Declared

